# Pulmonary aspergillosis in two COVID-19 patients from Kuwait

**DOI:** 10.1099/acmi.0.000201

**Published:** 2021-02-11

**Authors:** Khaled Alobaid, Beena Yousuf, Eman Al-Qattan, Zainab Muqeem, Nawaf Al-Subaie

**Affiliations:** ^1^​ Mycology reference laboratory, Mubarak Al-Kabeer hospital, Jabriya, 46307, Kuwait; ^2^​ Department of Intensive Care, Adan Hospital, Hadiya, 64020, Kuwait; ^3^​ Department of Microbiology, Adan Hospital, Hadiya, 64020, Kuwait; ^4^​ Department of Microbiology, Jaber Al Ahmad Hospital, South Surra, 23385, Kuwait; ^5^​ Department of intensive care unit, Ahmadi Hospital, Ahmadi, 61008, Kuwait

**Keywords:** COVID19, pulmonary aspergillosis, ARDS, Middle East, Kuwait

## Abstract

Critically ill patients with coronavirus disease 2019 (COVID-19) are at risk of invasive pulmonary aspergillosis. The clinical manifestations of a superimposed fungal infection in those patients are difficult to underpin. This is compounded by the non-specific radiological findings associated with this infection and the challenges associated with performing bronchoscopy in COVID-19 patients. We would like to present two COVID-19 cases who developed secondary *Aspergillus* pulmonary infection in the intensive care unit as shown by respiratory cultures, serum galactomannan and radiological images. Despite advanced intensive care and use of antifungal agents, both patients died eventually. This report illustrates the negative impact of secondary Aspergillosis and calls for the need of increased awareness of COVID19-associated pulmonary aspergillosis.

## Introduction

A novel coronavirus, severe acute respiratory syndrome coronavirus 2 (SARS-CoV-2), has led to a worldwide pandemic with devastating consequences in both developed and underdeveloped countries. COVID-19-related respiratory failure, requiring mechanical ventilation, has been associated with poor outcome [1]. Many of the interventions utilized in those patients, such as steroids, immunomodulatory agents, renal replacement therapy and extracorporeal membrane oxygenation (ECMO) predispose to nosocomial infections. Invasive pulmonary aspergillosis has recently been recognized as a major threat to critically ill patients with COVID-19 [2, 3]. In this case report, we describe two patients with severe COVID-19 who developed secondary pulmonary aspergillosis during their intensive care unit (ICU) stay and subsequently died despite maximal support. We aim to raise awareness of COVID-19 associated pulmonary aspergillosis (CAPA) and highlight some of the key diagnostic characteristics and therapeutic interventions.

## Cases description

### Patient A

A previously healthy patient in their forties, was seen in the emergency room (day 0) with worsening respiratory symptoms, which started 4 days prior to admission. He was recently diagnosed as COVID-19 using real-time PCR (qPCR) (Thermo Fisher Scientific, USA). The patient was conscious and alert but hypoxic on room air with oxygen saturation (SpO_2_) of 85 %, which improved to 92 % on 15 litres/minute (l min^−1^) oxygen via a non-rebreathing mask. The respiratory rate was 25 breaths/min, temperature of 38.8 °C, blood pressure (BP) of 113/62, and heart rate (HR) of 78/min. Initial chest x-ray revealed bilateral lung infiltrates. He was transferred to the ICU, where he was managed with high-flow nasal oxygen therapy (HFNO) and conscious proning. Lopinavir/ritonavir, hydroxychloroquine and piperacillin/tazobactam were started. Due to severe acute respiratory distress syndrome (ARDS) according to the Berlin definition [4] (day 8), the patient was intubated, sedated and mechanically ventilated. Over the next 2 days, he had multiple episodes of hypoxia managed with lung recruitment manoeuvres, proning and inhaled nitric oxide. There were no positive cultures, but due to worsening clinical condition, antibiotics were escalated to meropenem, and steroids were added (methyl prednisolone 2 mg/kg/day). Despite maximum ventilatory support, patient respiratory function continued to deteriorate. On day 12, the patient was placed on ECMO. Over the next 2 weeks, patient was managed on full ECMO support without any signs of lung recovery, so bronchoscopy was performed (day 25), which revealed highly inflamed and fragile mucosa. Bronchoscopic alveolar lavage (BAL) was obtained for microbiological investigation. It was inoculated into the following media: blood, chocolate, MacConkey and Sabouraud agar plates (Thermo Fisher Scientific, UK).

**Fig. 1.**
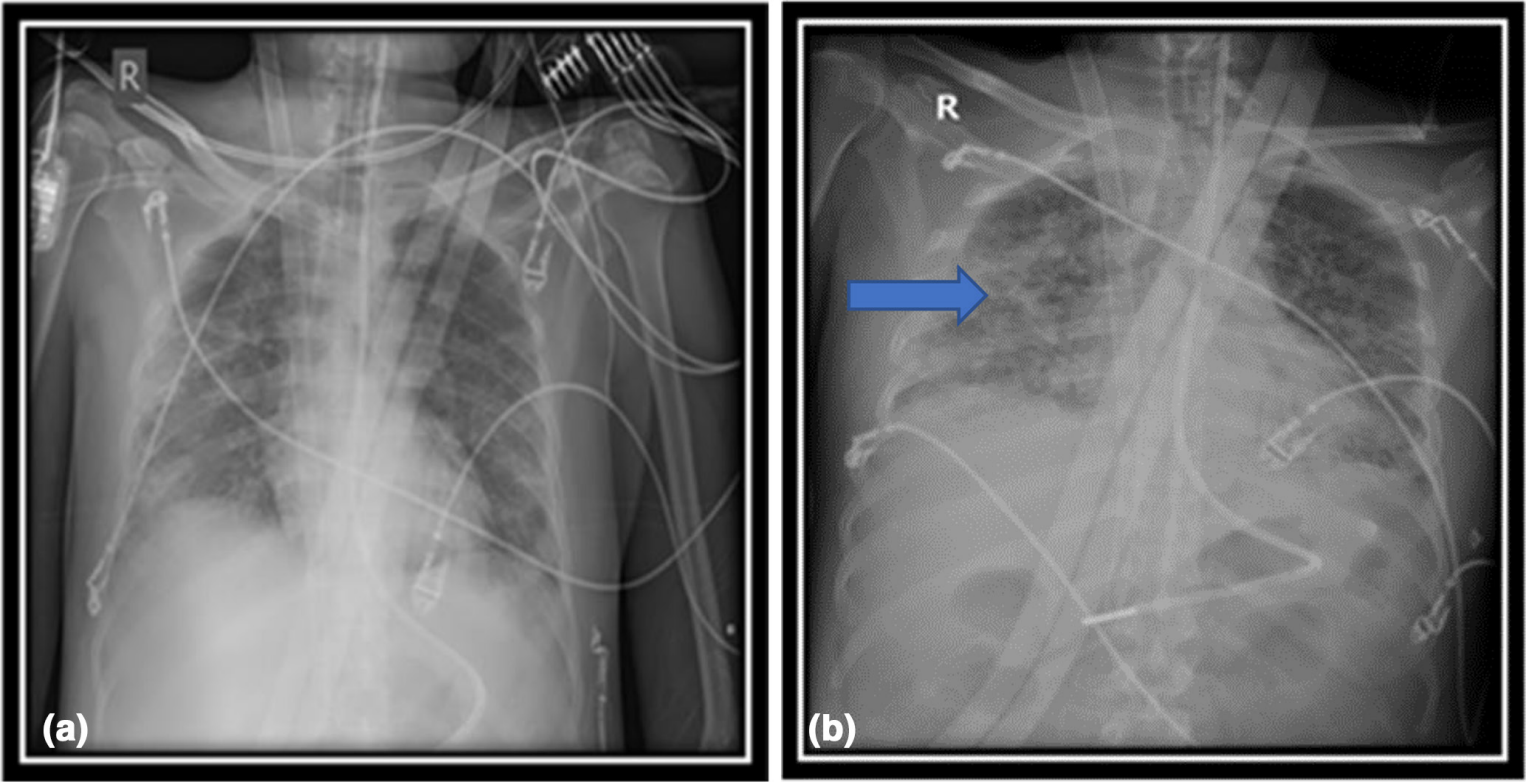
(a) One week before fungal infection (b) Diffuse opacification worse in the mid and lower zones of the right lung field. ECMO cannula positioned in the vena cava.

Microscopy revealed septate hyphae and accordingly, the patient was started on caspofungin 70 mg followed by 50 mg/day. The chest x-ray (CXR) showed worsening of lung infiltrates (Fig. 1). The BAL culture grew *Aspergillus niger,* which was identified by typical phenotypic features; colonies were granular with black coloured surface and pale to yellow reverse. Microscopically, phialides radiate from the entire vesicle, conidiogenous cells were biseriate and brown in colour. Antifungal susceptibility testing was performed by E test (bioMérieux SA, Marcy l'Etoile, France). MIC results in µg ml^−1^ were as follows: amphotericin B: 0.032, itraconazole: 0.38, caspofungin: 0.004 and voriconazole: 0.023. Subsequently the patient was switched (day 32) to voriconazole 400 mg twice a day followed by 200 mg twice a day via a nasogastric tube due to impaired renal function. The patient continued to be fully dependent on ECMO with no signs of respiratory recovery up to day 34. Few days later (day 37), the patient developed a methicillin-resistant *staphylococcus aureus* blood-stream infection, which he received vancomycin for. On day 41, there was an improvement in lung compliance as well as CXR appearance, and gradual weaning of sedation and ECMO support was attempted. However, the patient developed septic shock and multiorgan failure due to *Pseudomonas aeruginosa* and *Enterobacter aerogenes* bacteremia. The patient's condition continued to deteriorate and he died on day 53 following his hospital admission.

### Patient B

**Fig. 2.**
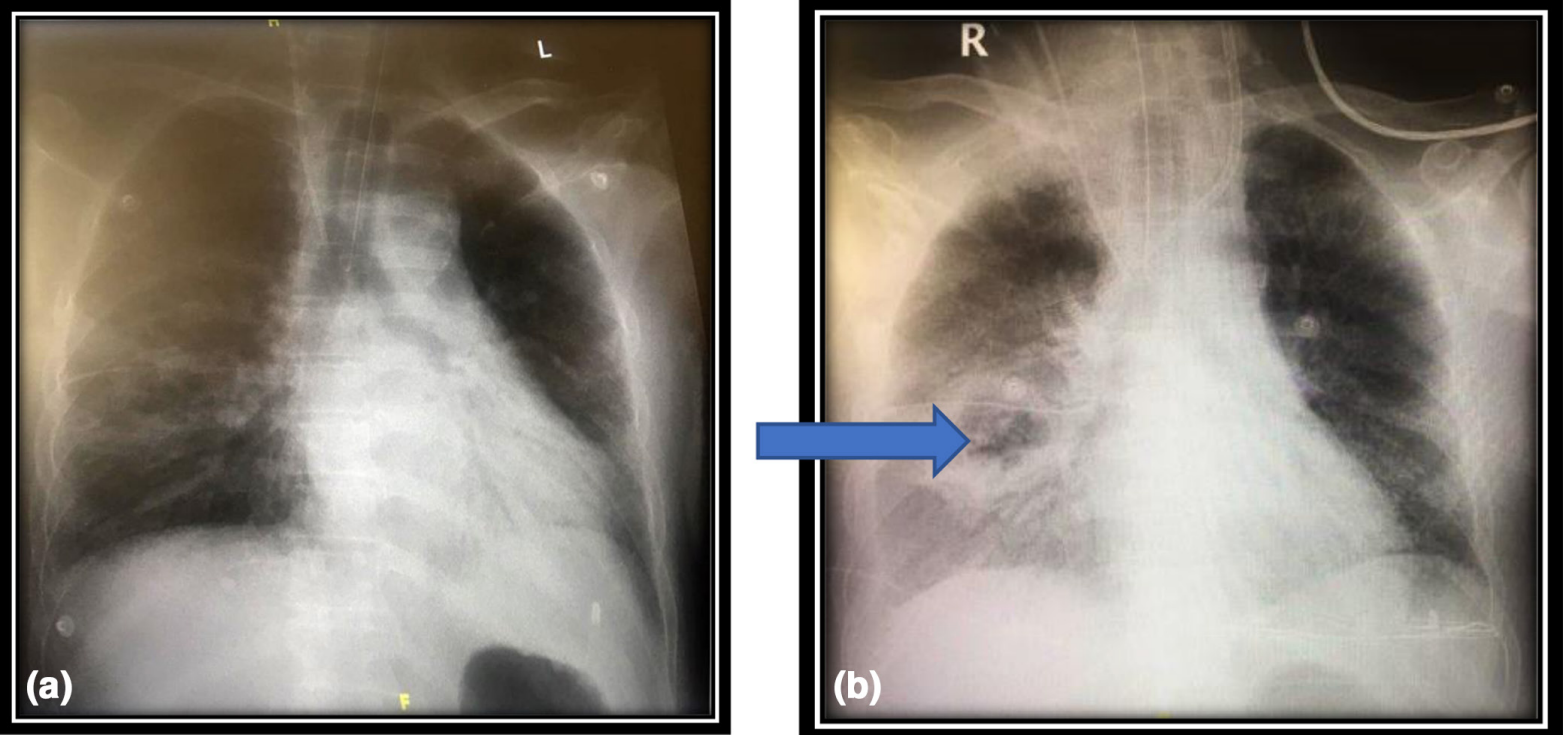
(a) One week before fungal infection. (b) Large solitary cavitating lesion involving the mid and lower zones of the right lung.

A patient in their sixties, who suffered from diabetes mellitus, hypertension and ischaemic heart disease, was referred to a secondary hospital (day 0) with COVID-19 pneumonia. On physical examination, the patient was tachypnic with a respiratory rate of 28 breaths/min and SpO_2_ of 86 % on 15 l min^−1^ oxygen via face mask. The patient was conscious, afebrile and haemodynamically stable. The CXR showed diffuse bilateral lung infiltrates more pronounced in the mid and lower zones. The patient was treated with piperacillin/tazobactam 4.5 g TID and levofloxacin 500 mg OD. (ceftriaxone, azithromycin and oseltamivir were administered 4 days prior to hospital admission). In view of his respiratory distress, the patient was transferred to the ICU, where he was connected to HFNO. The next day (day 1), hydroxychloroquine was added 400 BID, then 200 BID. The patient also received a single dose of tocilizumab 600 mg IV. On day 9, he started to improve from a respiratory point of view and was consequently able to move out of bed for the first time. However, 2 days later (day 14), he developed septic shock and hypoxia (SpO_2_ 84 %) and was eventually intubated and mechanically ventilated. The CXR showed progression in the previously noted bilateral infiltrates. Blood cultures revealed *Klebsiella pneumoniae *, which was treated with meropenem 500 mg BID. In addition, anidulafungin (200 mg then 100 mg OD) and hydrocortisone (50 mg 4 hourly for 4 days) were added (day 16). In the following days, he was still requiring mechanical ventilation, but showed some improvement in his respiratory parameters. However, on day 23, the patient developed acute kidney injury requiring renal replacement therapy. On day 26, an endotracheal aspirate grew *Aspergillus niger*. Antifungal susceptibility testing done by E test (bioMérieux SA, Marcy l'Etoile, France) revealed the following MICs (µg ml^−1^): amphotericin B: 0.125, itraconazole: 0.032, caspofungin: 0.002 and voriconazole: 0.094. In addition, his CXR has revealed a large cavitating lesion in the right lung (Fig. 2). Accordingly, the patient was switched to liposomal amphotericin B 350 mg OD. A serum galactomannan test (Platelia *Aspergillus* EIA, Bio-Rad) was positive (6.53). Despite all supportive measures and ongoing antimicrobial therapy, the patient developed multiorgan failure and died eventually (day 31).

## Discussion

The ongoing pandemic of SARS-CoV-2 continues to cause considerable morbidity and mortality. In Kuwait, at the time of writing, the total number of cases has reached 131 743 cases, with subsequent 811 deaths [5]. This report describes two cases of pulmonary aspergillosis among COVID-19 patients from two different hospitals in Kuwait. The list of reported CAPA cases is growing rapidly worldwide [6–8]. While this clearly indicates that CAPA is not geographically restricted, epidemiology may differ across different countries due to the diverse clinical practices in ICUs and the different modalities used to treat COVID-19-infected patients. The true prevalence of CAPA remains unknown, but according to several observational studies, it varies widely from 4–35 % as shown in Swiss and Belgian studies, respectively [2, 8]. Although there is currently no clear definition for CAPA, Verweij and colleagues have proposed a definition for influenza-associated pulmonary aspergillosis [9] that has been recently validated for CAPA by a prospective multicentre study [10]. Using this definition, both cases presented are classified as probable pulmonary aspergillosis.

Unlike influenza-associated aspergillosis, where the onset of pulmonary aspergillosis occurs early in the course of the disease (median 3 days from ICU admission), our patients developed CAPA much later (~day 25 post-ICU) [11]. Other studies have shown variable timing from the date of ICU admission and the development of pulmonary aspergillosis among COVID-19 patients [3]. This variability could be explained by the frequency and timing of mycological testing. A clear example is shown in patient B, where the respiratory culture was collected days after clinical deterioration.

The microbiologic diagnosis of *Aspergillus* infection was confirmed by a positive culture from a BAL specimen in patient A, while patient B had a positive endotracheal aspirate culture accompanied by positive serum galactomannan test. Bronchoscopy can aid in the diagnosis of CAPA by assessment of the tracheobronchial tree for evidence of tracheobronchitis and for obtaining BAL samples for mycological analysis [3, 12]. However, this is not always feasible due to the possibility of aggravating the patient’s respiratory failure and the risk of aerosolization. In such cases, endotracheal aspirate samples are usually taken, even though they offer low sensitivity and fail to discriminate colonization from infection. Serum galactomannan is an attractive option, but lacks sensitivity in CAPA cases, but can be helpful in establishing diagnosis early when the test is positive [3, 13]. On the other hand, BAL galactomannan testing is more sensitive in diagnosing pulmonary aspergillosis and is recommended for the diagnosis of CAPA [3, 6, 12]. Glucan test is a serum test that may play a role in diagnosing invasive aspergillosis in an ICU setting. However, negative test may not rule out the infection, and more studies are needed to determine the performance of such test specifically in CAPA patients [3]. Besides fungal biomarkers, *Aspergillus* PCR can also be used. In a recent review of 38 published CAPA cases, *Aspergillus* DNA was detected in serum or BAL specimens from 13 out of 19 patients [6]. In contrast to the majority of CAPA cases reported so far, where the main etiologic agent was *Aspergillus fumigatus*, our patients were both infected with *Aspergillus niger*. The same species has also been reported in Pakistan [7].

In terms of radiologic findings, chest computerized tomography (CT) scan was not performed at the time of positive fungal culture, but serial CXR films for patient A have revealed worsening pulmonary infiltrates, while the CXR image of patient B has shown a cavitating lesion. Of note, the radiological findings of invasive pulmonary aspergillosis are usually non-specific among non-neutropenic patients [12].

While both patients were not immune-compromised, they have received steroids before the onset of CAPA. Steroids have recently been shown to improve survival among COVID19 patients [14], but such use may also predispose to invasive aspergillosis [15]. Beside steroid therapy, patient A was on ECMO, which increases the risk of opportunistic infections including invasive pulmonary aspergillosis [16]. On the other hand, patient B had received tocilizumab, an IL-6 inhibitor, which is postulated to play a role in modulating the cytokine storm seen in severe COVID19 cases [17]. However, this may compromise the immune system particularly helper T-cell (Th17) responses, and hence may predispose to CAPA [18]. In fact, the high incidence of invasive aspergillosis observed in the first prospective CAPA study is possibly due to the wide usage of steroids and tocilizumab [10]

With regard to anti-fungal treatment, both patients had received caspofungin initially, then shifted to voriconazole and liposomal amphotericin B in patients A and B, respectively. Ideally, the first-line therapy for invasive pulmonary aspergillosis should be intravenous voriconazole or isavuconazole. To ensure optimal therapeutic concentration, voriconazole serum trough level should be carefully monitored [19].

In conclusion, this report attempts to increase the awareness of invasive pulmonary aspergillosis in COVID19 patients with ARDS. Appropriate and timely use of mycological tests, and better communication between different specialties such as intensivists, radiologists and microbiologists are strongly encouraged.
